# Trypacidin, a Spore-Borne Toxin from *Aspergillus fumigatus*, Is Cytotoxic to Lung Cells

**DOI:** 10.1371/journal.pone.0029906

**Published:** 2012-02-03

**Authors:** Thierry Gauthier, Xiaodi Wang, Joice Sifuentes Dos Santos, Athanasios Fysikopoulos, Souria Tadrist, Cécile Canlet, Marie Pierre Artigot, Nicolas Loiseau, Isabelle P. Oswald, Olivier Puel

**Affiliations:** UMR1331 TOXALIM, French National Institute for Agricultural Research, INP, UPS, Toulouse, France; David Geffen School of Medicine at University of California Los Angeles, United States of America

## Abstract

Inhalation of *Aspergillus fumigatus* conidia can cause severe aspergillosis in immunosuppressed people. *A. fumigatus* produces a large number of secondary metabolites, some of which are airborne by conidia and whose toxicity to the respiratory tract has not been investigated. We found that spores of *A. fumigatus* contain five main compounds, tryptoquivaline F, fumiquinazoline C, questin, monomethylsulochrin and trypacidin. Fractionation of culture extracts using RP-HPLC and LC-MS showed that samples containing questin, monomethylsulochrin and trypacidin were toxic to the human A549 lung cell line. These compounds were purified and their structure verified using NMR in order to compare their toxicity against A549 cells. Trypacidin was the most toxic, decreasing cell viability and triggering cell lysis, both effects occurring at an IC_50_ close to 7 µM. Trypacidin toxicity was also observed in the same concentration range on human bronchial epithelial cells. In the first hour of exposure, trypacidin initiates the intracellular formation of nitric oxide (NO) and hydrogen peroxide (H_2_O_2_). This oxidative stress triggers necrotic cell death in the following 24 h. The apoptosis pathway, moreover, was not involved in the cell death process as trypacidin did not induce apoptotic bodies or a decrease in mitochondrial membrane potential. This is the first time that the toxicity of trypacidin to lung cells has been reported.

## Introduction

For over twenty years, *Aspergillus fumigatus* has been considered as the most common airborne fungal pathogen. It is a saprophytic fungus that can grow outdoors on different organic materials including cereals, malted barley, packed hay, compost and tobacco. Growth indoors has been reported using water-damaged building materials [1], old paper [2] and damp wallpaper [3] as substrates. Exposure to inhaled *A. fumigatus* spores is, therefore, likely common throughout life. The size of such spores (2 to 3 µm in diameter) allows them to travel easily through the respiratory tract and into lung alveoli. The spores are commonly found in the environment and can exceed 10^4^ spores/m^3^ in damp buildings or even 10 to 100 times more in waste or compost handling facilities [4]. More than a hundred genotypes of *A. fumigatus* can be present in hospital buildings [5]. This fungus causes different pathologies including occupational rhinitis in people working in damp and mouldy places [6], allergic aspergillosis and invasive forms of aspergillosis in immunosuppressed patients [7]. Several cases of acute pulmonary aspergillosis in immunocompetent patients have been reported [8], [9], [10]. The factors that enable *A. fumigatus* to cause invasive disease are not currently understood [11].

Previous studies had identified the presence of 26 gene clusters in the *A. fumigatus* genome putatively involved in secondary metabolism [12], [13]. Using the Smurf software, we could update the number of clusters to 28 and 30 for A1193 and Af293 strain respectively. These data suggest a major production of secondary metabolites by *A. fumigatus* [14]. The chemical structures of a large number of metabolites are well characterized including the well-known gliotoxin [15], which has multiple cytotoxic and immunosuppressive properties [11]. Gliotoxin is the only toxin isolated from the sera of rodents and of patients suffering from invasive aspergillosis [16]. The contribution of gliotoxin *in vivo* was characterized by disrupting either the gene encoding the non-ribosomal peptide synthase *gliP* or the gene encoding the transcriptional factor *gliZ*, both of which are indispensable to gliotoxin biosynthesis. The decrease in virulence of mutant strains, however, has only been demonstrated in non-neutropenic mice suggesting that gliotoxin plays an important role in the pathogenesis in immunosuppressed but not neutropenic patients [17]. The use of HPLC and TLC, furthermore, failed to detect gliotoxin in spores [18], while it is well known that gliotoxin is produced by the fungus once it is growing within the target tissue [7], [11].

Although the gliotoxin toxicity and its putative involvement in *A. fumigatus* mycoses have been well documented, few data regarding the potential toxicity of other metabolites present on the conidia are available. Fumigaclavine C and three other minor ergot alkaloids, festuclavine, fumigaclavine A and fumigaclavine B have also been shown to be associated with *A. fumigatus* conidia [19]. It has been shown that conidia from *A. fumigatus* grown on different media bear other metabolites including trypacidin, tryptoquivaline, fumitremorgin A [20]. Moreover, only trypacidin and tryptoquivaline were found in the extract of bioaerosols from compost facilities containing 3.2×10^7^ *A. fumigatus* spores/m^3^ [21]. Trypacidin and monomethylsulochrin were isolated from *A. fumigatus* at the beginning of the 1960 s [22], [23]. The antibiotic and antiprotozoal properties of trypacidin were described in 1963 [22] and the chemical structure was identified in 1965 [24]. No attempt has been made so far to evaluate the toxicity of these metabolites on the respiratory tract.

The objectives of the present study were, therefore, to evaluate the toxicity of the metabolites borne by the spores of *A. fumigatus* and to determine the cytotoxic effects they induce in lung cells.

## Materials and Methods

### Chemicals

Gliotoxin, verruculogen, fumagillin and helvolic acid were purchased from Sigma-Aldrich (Saint Quentin Fallavier, France), fumitremorgin C, pseurotin A and fumigaclavine A from Alexis Biochemicals (Enzo Life sciences, Farmingdale, NY), sulochrin from BioAustralis Fine Chemicals (Smithfield, Australia). All commercial products were used without further purification (purities higher than 90%). The sources of the other secondary metabolites used as authentic standards were: J.W Dorner, National Peanut Research Laboratory, ARS-USDA, Dawson, USA (fumigaclavine C), C. Avendano, Universidad Complutense, Madrid, Spain (fumiquinazoline F), and P.G. Mantle, Imperial College, London, UK (TR2 toxin). All solvents used in extraction, flash chromatography, liquid chromatography-diode array detector (LC-DAD) and liquid chromatography-mass spectrometry (LC-MS) were analytical grade. Water for high performance liquid chromatography was purified by using a Millipore MilliQ purification system.

### Metabolite extraction from conidia

Large amounts of conidia from *Aspergillus fumigatus* NRRL 35693 strain were produced on Czapek yeast extract agar (CYA) and potato dextrose agar (PDA) medium for 14 days at 37°C in the dark. Ten 90 mm plates were prepared for each culture medium. The conidia were harvested as described previously [25]. Then, the conidia solution was filtered through a porosity 2 filter funnel (Robu, Hattert, Germany) following a second filtration through Miracloth (Merck Chemicals, Darmstadt, Germany) to remove hyphae. The absence of mycelium was checked by optical microscopy.

### Fungal strain culture conditions

Fifteen other fungal strains of *A. fumigatus* from the Pharmacology-Toxicology Laboratory New Collection (NCPT) were examined for their ability to produce trypacidin (Table S1). The strains were selected from various human, animal, plant and environmental sources and geographical regions. In order to determine which mycotoxins are associated with conidiogenesis, we compared HPLC-DAD and MS analyses of culture extracts from the conidiation-deficient *A. fumigatus* strain (Δ*brlA) vs* the conidiation-restored strain (Δ*brlA::brlA)*. The generation and morphological characterization of both strains have been described previously [26]. The strains were grown on PDA for 8 days at 25°C and the cultures then extracted with 70 ml chloroform. After filtration through Whatman SP filters, the organic phase was evaporated under vacuum at 50°C using a rotary evaporator. The residue was taken up in 200 µl of methanol and this suspension filtered through a 0.45 µm disposable filter before HPLC analysis. Trypacidin production by *A. fumigatus* strains was measured after culture on autoclaved grains of CapHorn, a variety of common wheat. Wheat grains were moistened with sterile distilled water for 4 days at 4°C (to reach a water activity a_W_ of 0.98) before 20 min sterilization at 120°C. Thirty grams were placed into a 140 mm diameter petri dish and inoculated with 250 µl of a spore suspension containing 2×10^5^ conidia, obtained from a preliminary culture on PDA medium at 37°C for 7 days. The cultures on the grains were incubated at 25°C for 13 days. The *A. fumigatus* NRRL35693 (NCPT13) strain was used to identify the toxic metabolites produced by the fungus and to produce and purify trypacidin, monomethylsulochrin and questin.

### HPLC-MS and MS-MS analysis of fractions

Chromatographic analyses were performed on a Thermo-Finnigan surveyor HPLC system with a diode array detector (DAD) and a LCQ XP MAX® ion trap mass spectrometer (Thermo Electron Corporation, Waltham, MA). The LC separation was accomplished with a 150 mm×2.1 mm Luna 5 µm C18 column (Phenomenex, Torrance, CA). A gradient elution was used at a flow rate of 0.2 ml/min with a mobile phase of 16.6 mM acetic acid (A) and acetonitrile (B) under the following conditions: the program initiated with 80/20% (v/v) eluent A/B for 5 min, then eluent B was increased to 50% (v/v) over 25 min, and to 90% (v/v) over 5 min, held constant at 90% (v/v) for 10 min, then finally returned to the initial 20% (v/v) and kept at this level for the last 10 min. Molecules were ionized with an electrospray ionization source in negative (ESI−) and positive mode (ESI+). For the negative mode, the spray needle was set at a potential of 4.5 kV. Capillary voltage and temperature were −15 V and 375°C respectively. For the positive mode, the source voltage was increased to 5 kV whereas capillary voltage was set to 3 V. The capillary temperature remained unchanged. For both modes, sheath gas and auxiliary gas flow rate of nitrogen were at 70 and 40 (arbitrary units). The tube lens offsets were set for negative and positive mode to −45 V and 5 V respectively. Helium was used in the trap as the damping and collision gas. Culture and fraction extracts were analysed in full scan and MS/MS mode with a *m*/*z* range of 50 to 800. MS and MS-MS tuning were performed on authentic fumigaclavine C and the MS-MS collision was set at 40%.

### Fractionation of culture extracts

After extraction of secondary metabolites with chloroform, the raw extract was fractioned by an automated flash chromatography instrument (Teledyne Isco, Lincoln, NE). The fractionation was performed on a normal phase RediSep 12 g column (Teledyne Isco, Lincoln, NE) at a flow rate of 25 ml/min with a linear gradient. The solvent system was composed of 10% acetic acid in toluene as solvent A and 10% acetic acid in ethyl acetate as solvent B. The % of solvent B increased from 0% to 45% within 10 min with a fraction volume of 5 ml. Thirty fractions were collected from the sample extracted in chloroform. The most active fraction was evaporated, dissolved in methanol and fractionated by HPLC-DAD using a 150 mm×4.6 mm Luna C18 5 µm column (Phenomenex, Torrance, CA) in a HPLC chromatograph (Kontron, Milan, Italy). The flow rate was set to 1.5 ml/min. A gradient of water with 0.1% acetic acid and acetonitrile was used as mobile phase increasing from 10% to 40% acetonitrile in 18 min, then increasing to 90% within 10 min, maintained for 10 min, before returning to the initial proportions. The fractionation was performed as follows: F1 (1–10 min), F2 (10–15 min), F3 (15–17 min), F4 (17–19 min), F5 (19–21 min), F6 (21–23 min), F7 (23–25 min), F8 (25–30 min), F9 (30–40 min) and F10 (40–46 min).

### Purification of secondary metabolites

Trypacidin, monomethylsulochrin and questin were purified from the crude extract by a two step semi-preparative HPLC. In the first step, a 250 mm×7.8 mm Modulo-Cart Strategy 5 µm C18 column (Interchim, Montluçon, France) was used under isocratic conditions (32% (v/v) acetonitrile, 68% (v/v) 16.6 mM acetic acid) with a flow rate of 4.5 ml/min. Samples enriched in trypacidin and in monomethylsulochrin and questin were obtained. In the second step, the mobile phase was methanol and 16.6 mM acetic acid with a flow rate of 3.5 ml/min. Trypacidin containing samples were chromatographed isocratically with 45% (v/v) methanol and the monomethylsulochrin and questin containing samples at 50% (v/v) methanol. Purified fractions were evaporated before being dissolved in methanol.

The purity of the compounds was confirmed by LC-MS and NMR as described below. The concentration of the samples was measured by reading the absorbance at 286 nm for trypacidin (ε_25°MeOH_ = 23 400 M^−1^ cm^−1^) and questin (ε_25°MeOH_ = 25 704 M^−1^ cm^−1^) and 285 nm for monomethylsulochrin (ε_25°MeOH_ = 14 700 M^−1^ cm^−1^).

### Nuclear Magnetic Resonance

^1^H and two-dimensional nuclear magnetic resonance (2D-NMR) spectra were generated using a Bruker Avance DRX-600 spectrometer (Bruker, Wissembourg, France) operating at 600.13 MHz in CDCl_3_ solution (70–200 µg sample/600 µl solvent in a 5 mm NMR tube).

All measurements were obtained using a Bruker cryoprobe TXI at 300 K with tetramethylsilane as reference standard.

To confirm the chemical structures, samples were analyzed using ^1^H and 2D-NMR including gradient selection (gs)-correlation spectroscopy (COSY), (gs)-heteronuclear single quantum coherence (HSQC) and (gs)-heteronuclear multiple bonding connectivity (HMBC).

^13^C chemical shifts were determined from the f1 projection of HSQC and HMBC diagrams, because the low amount of product (70 µg) precluded the direct measurement of a carbon spectrum.

The spectral data of the analysed compounds (questin, monomethylsulochrin, trypacidin, fumiquinazoline C, tryptoquivaline F) are described in Figure S1.

### Cell culture conditions

The human alveolar carcinoma cell line A549 was obtained from the DSMZ-German Collection of Microorganisms and Cell Cultures (Braunshweig, Germany). Cells were cultured in Dulbecco's Modified Eagle Medium (DMEM) with added 10% heat-inactivated fetal calf serum, 2 mM L-glutamine, 100 IU/ml penicillin and 100 µg/ml streptomycin at 37°C in a humidified atmosphere with 5% CO_2_.

The human bronchial epithelial cells (HBEpC) were purchased from Cell Application Inc. (San Diego, CA). HBEpC are primary cells derived from normal human bronchi. They were cultured in Bronchial Epithelial Cell Growth Medium provided by Cell Application Inc. at 37°C in a humidified atmosphere with 5% CO_2_.

### Cell viability measurement: MTT assay

Cell viability was determined using the CellTiter 96® Non- Radioactive Cell Proliferation Assay (Promega, Madison, WI). The assay is based on the cellular conversion of a tetrazolium salt (3,(4,5-dimethylthiazol-2-yl) 2,5-diphenyltetrazolium bromide) into a formazan product by mitochondrial succinate dehydrogenase. The formazan product is detected by absorbance at 570 nm. Cells were plated into 96-well flat plates in 100 µl growth medium (1×10^4^ cells/well for A549 and 2×10^4^ cells/well for HBEpC) and cultured for 24 h. The cells were then exposed to toxins for either 24 h or 48 h. Negative and positive controls were obtained by treating cells with respectively the solvent alone or with 10 µM gliotoxin. Blanks were obtained with the absence of cells in the wells. A 3,(4,5-dimethylthiazol-2-yl) 2,5-diphenyltetrazolium bromide (MTT) solution (5.0 mg/ml) was added to the wells (15 µl/well), and the plates incubated at 37°C in 5% CO_2_ for 4 h. The absorbance (Abs) was measured in a microplate reader ‘Infinite 200’ (Tecan, Grödig/Salzburg, Austria) at 570 nm and 650 nm (reference). The % viability was calculated as follow: (Abs_toxin_ – Abs_blank_)/(Abs_negative_ – Abs_blank_) ×100%.

The toxin concentration needed to reduce cell viability by 50% (IC_50_) was calculated using the software SigmaPlot (Systat Software Inc., San Jose, CA).

### Cell lysis measurement: LDH assay

Cell lysis was measured using the CytoTox 96® Non-Radioactive Cytotoxicity Assay (Promega, Madison, WI). 1×10^4^ A549 cells/well were plated into 96-well round bottom plates in 100 µl growth medium for 24 h and then exposed to toxins for another 24 h. Negative and positive controls were obtained by treating cells with respectively the solvent alone or with 50 µM gliotoxin. After centrifugation at 450×g for 5 min, 30 µl of cell culture medium were collected and placed in a 96-well microtitre plates. The cells were then treated with 70 µl 1.8% Triton X-100 for 30 min at 37°C to release the total amount of LDH and then 30 µl were collected and placed in another 96-well microtitre plate. Fifty µl of the substrate solution were added and after thirty min the reaction was stopped by adding 70 µl of 1N acetic acid. Absorbance at 490 nm was recorded to measure formazan production which correlated with the amount of LDH in the wells. The ratio between the LDH released in the supernatant and the total amount of LDH indicates the percentage of lysis triggered by the toxin. The toxin concentration needed to cause 50% cell lysis (IC_50_) was calculated using the software SigmaPlot (Systat Software Inc., San Jose, CA).

### Cell cycle and apoptosis assay

The cell cycle was investigated using DNA flow cytometry [27]. A549 or HBEpC cells (3×10^5^ cells/well) were seeded in 12-well plates and incubated until reaching approximately 70% confluence. The cells were then exposed to different concentrations of trypacidin (1, 10, 30, 50 µM) or gliotoxin 5 µM for 24 h. Cells were harvested after trypsinization and centrifugation at 450×g for 6 min, washed with phosphate-buffered saline (PBS) and then fixed in 75% (v/v) ethanol for 4 h at −20°C. The cells were collected by centrifugation, washed twice with PBS and suspended in 0.4 ml of a propidium iodide solution containing RNase (BD Pharmingen, San Diego, CA) for 30 min at 37°C. Flow cytometry was used to sort cells in G_0_/G_1_ phase (2 n DNA), S phase (between 2 n and 4 n DNA), G_2_/M phase (4 n DNA) and sub-G1 particles (<2 n DNA). The stained cells were analysed using a flow cytometer (Becton Dickinson FACSCalibur System, San Jose, CA) and the data were calculated using Flowjo software (Tree Star, Inc., Ashland, OR).

### Measurement of the mitochondrial transmembrane potential (ΔΨm)

The mitochondrial transmembrane potential was assessed using 3,3′-dihexyloxacarbocyanine iodide (DiOC_6_, Molecular Probes, Madison, WI) whose accumulation on the inner surface of the inner mitochondrial membrane is driven by ΔΨm. After 2 h and 24 h exposure to 10 and 50 µM trypacidin, the cells were harvested by centrifugation at 450×g for 6 min and incubated in 1 ml of cell suspension buffer (10 mM Hepes pH 7.4, 125 mM NaCl, 4.7 mM KCl, 1.2 mM KH_2_PO_4_, 1 mM MgCl_2_, 1 mM CaCl_2_, 10 mM glucose) containing 50 nM DiOC_6_ for 20 min at 37°C. Carbonylcyanide-4-(trifluoromethoxy)-phenylhydrazone 40 µM (FCCP, Enzo Life Sciences, Ann Arbor, MI) was used as positive control. The cells were then collected by centrifugation and suspended in 0.3 ml PBS. DiOC_6_ fluorescence was recorded using a flow cytometer (Becton Dickinson FACSCalibur System, San Jose, CA) and the data were calculated using Flowjo software (Tree Star, Inc., Ashland, OR).

### Detection of reactive oxygen species (ROS) and reactive nitrogen species (RNS)

A549 cells were treated with 10 µM and 50 µM trypacidin for 1, 2 and 24 h in the presence of oxidation-sensitive fluorescent probes: 5 µM dihydroethidium (DHE, Molecular Probes, Madison, WI) to detect superoxide anion (O_2_^•−^), 10 µM dichlorodihydro-fluorescein diacetate (H2DCF-DA, Molecular Probes, Madison, WI) to detect H_2_O_2_ and 5 µM 4-amino-5-methylamino-2′,7′-difluorofluorescein diacetate (DAF-FM, Molecular Probes, Madison, WI) to detect NO. Pro-oxidants were used as positive controls: 100 µM menadione (Sigma-Aldrich, St. Louis, MO) for DHE and H2DCF-DA, and 2.5 mM S-nitroso-N-acetylpenicillamine (SNAP, Molecular Probes, Madison, WI) for DAF-FM. Dead cells were stained by adding 300 ng/ml propidium iodide (Molecular Probes, Madison, WI). Cells were harvested by centrifugation and suspended in 0.3 ml PBS before analysis using a flow cytometer (Becton Dickinson FACSCalibur System, San Jose, CA). The data were calculated using Flowjo software (Tree Star, Inc., Ashland, OR).

## Results

**Figure 1:**
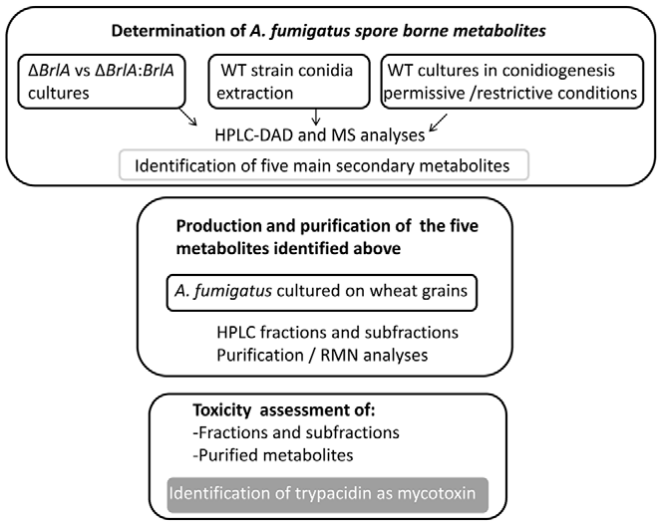
Overall strategy to assess the toxicity of spore borne metabolites.

The overall strategy of the current study is summarized in figure 1:

In a first step, the spore borne metabolites were identified by three independent experiments:

- Comparison of secondary metabolites produced from conidiogenesis deficient *Δbrla* strain and from reverse mutant Δ*brla::brlA* strain.
- Comparison of secondary metabolites produced when the wild type strain is cultured under conidiogenesis inducing conditions *vs* restrictive conditions.
- LC-MS analyses of conidia extract.

In a second step, the production and purification of the identified spore borne metabolites were achieved from *A. fumigatus* culture grown in the best conditions for sporulation. The purified metabolites were analyzed for their toxic effects on A549 lung cells.

### Identification of spore borne metabolites from *Aspergillus fumigatus*

**Figure 2:**
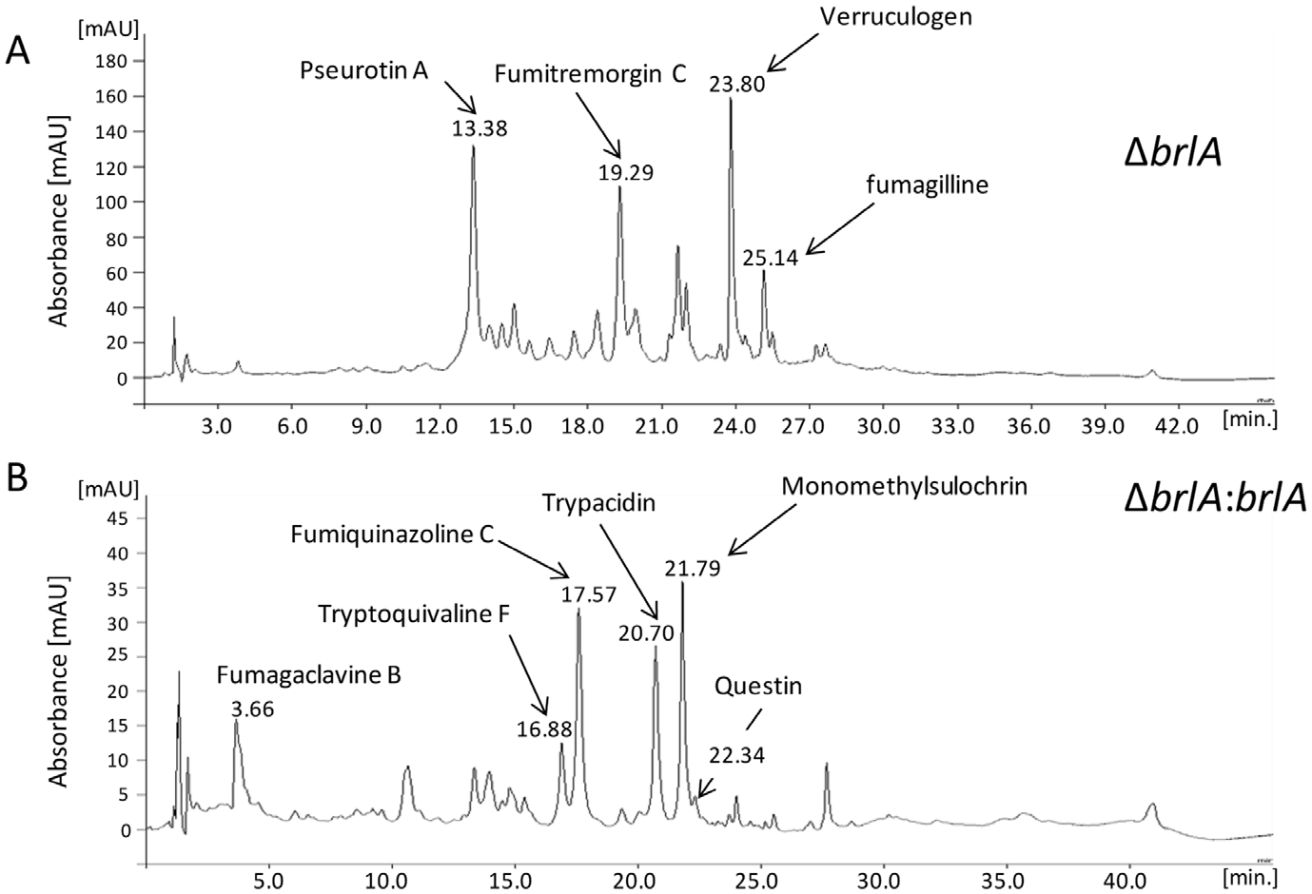
HPLC-chromatograms at 270 nm of *A. fumigatus* extracts. A) conidiation-deficient *A. fumigatus* strain (Δ*brlA)* and B) conidiation-restored strain (Δ*brlA::brlA)*. The fungi were cultured at 25°C on PDA for 13 days in the dark as described in the Materials and Methods.

**Figure 3:**
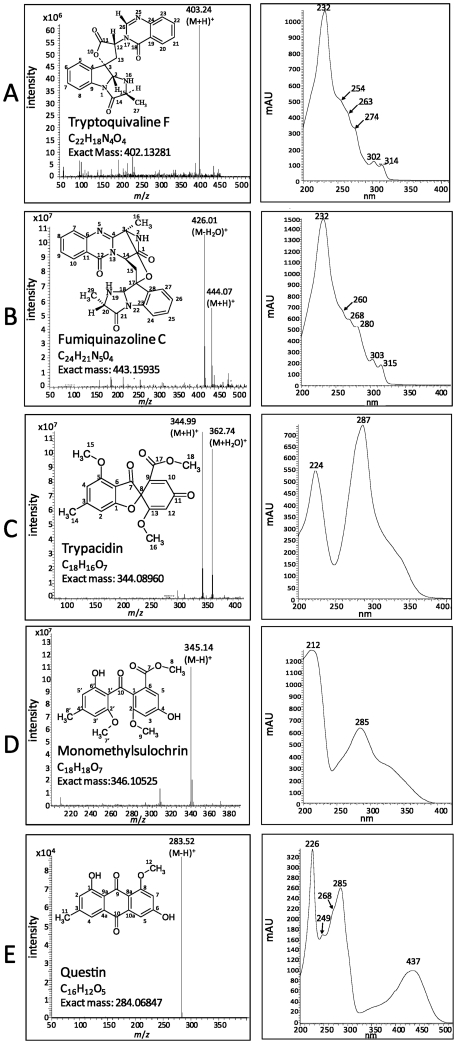
Identification and structures of the 5 major compounds isolated from *A. fumigatus* conidia extracts. MS spectra (left) and UV-Vis spectra (right). A) tryptoquivaline F/J, B) fumiquinazoline C, C) trypacidin, D) monomethylsulochrin, E) questin.

In order to determine which mycotoxins are associated with conidiogenesis, comparisons of HPLC-DAD and MS analyses of culture extracts from conidiation-deficient Δ*brlA* and conidiation-restored Δ*brlA*::*brlA* strains were made. In accordance with Coyle et al. [26], the conidiating strain produced fumigaclavine B and five other metabolites that were not detected in crude extracts of the Δ*brlA* mutant (Figure 2). The first eluted metabolite (16.88 min) showed a typical fumiquinazoline/tryptoquivaline UV spectrum and a base peak at *m*/*z* 403 in LC-MS positive mode. After collision, the MS^2^ peaks were observed at *m*/*z* 239 (100%), *m*/*z* 199 (90%), *m*/*z* 211 (60%), *m*/*z* 171 (%) and *m*/*z* 147 (10%). This metabolite was identified as tryptoquivaline F or J (Figure 3A). The second eluted metabolite (17.57 min) had the same UV spectrum but showed a base peak at *m*/*z* 444 in LC-MS positive mode. The collision-induced dissociation MS-MS spectrum at *m*/*z* 444 led to a fragment at *m*/*z* 199 (100%), *m*/*z* 228 (80%), *m*/*z* 373 (60%) and *m*/*z* 171 (20%). This metabolite was identified as fumiquinazoline C (Figure 3B). The compound eluted in the third position (20.70 min) gave a UV spectrum characteristic of trypacidin and showed a base peak at *m*/*z* 345 (Figure 3C). The MS^2^ peaks were observed at *m*/*z* 301 and *m*/*z* 313. The following metabolite (21.79 min) had the UV spectra of monomethylsulochrin and showed a base peak at *m*/*z* 345 in LC-MS negative mode (Figure 3D). Its MS^2^ spectra displayed *m*/*z* 313 (100%) and *m*/*z* 181 (40%). Finally, the last compound (22.34 min) gave a typical anthraquinone UV spectrum and showed a base peak at *m*/*z* 283 in LC-MS negative mode. These results strongly suggested that this product was questin (Figure 3E). The identity of all five products was confirmed by NMR analyses (Figure S1).

**Figure 4:**
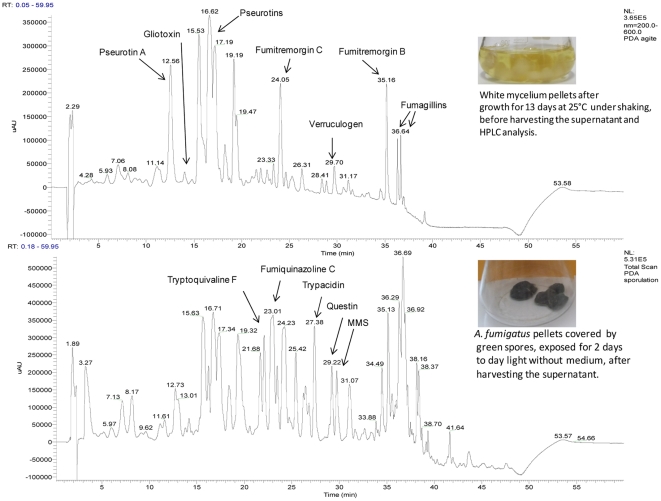
HPLC-DAD chromatograms from *Aspergillus fumigatus* (NRRL 35693) when grown in condition repressing (A) and inducing (B) conidiogenesis. The NRRL 35693 strain was cultured on Czapek-Glucose medium, at 25°C for 13 days in the dark under shaking (180 r.p.m.). Every day, the culture (mycelia and medium) was removed from the flask and placed in a new sterile flask in order to avoid that mycelium adhering to glass and sporulating in contact with the air. After 13 days, culture medium was harvested and analyzed by DAD-HPLC. The remaining pellets of mycelia were exposed to daylight without medium at 25°C in order to induce conidiogenesis. The secondary metabolites were extracted from pellets as described in the Materials and Methods.

Meanwhile, in a second experiment, the wild type strain (NRRL35693) was grown under condiogenesis repressive and inducing conditions. Then, the extract from culture was analysed by HPLC-DAD (Figure 4). As long as the fungus did not sporulate, many compounds were specifically detected (pseurotins, fumagillins, fumitremorgins, verruculogen and gliotoxin) but none of the five products identified above. However, two days after conidiogenesis induction, chromatography analyses revealed the presence of fumiquinazoline C, tryptoquivaline F, trypacidin, questin and monomethylsulochrin. Although both experiments showed that these five metabolites are associated to conidiation their location on the conidia still remained to be proved.

**Figure 5:**
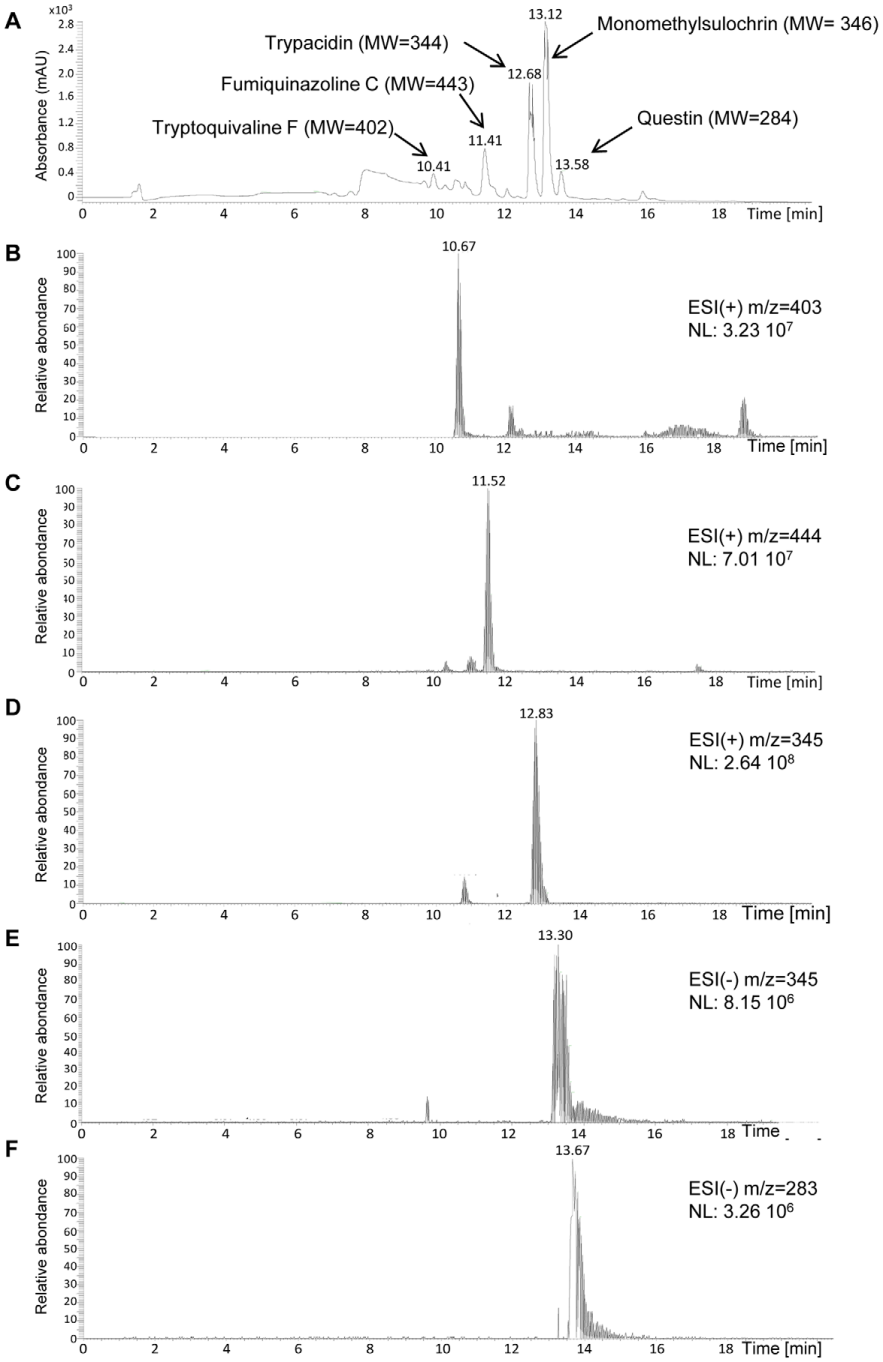
Representative HPLC-DAD and LC-MS chromatograms of conidial extract from *A. fumigatus*. *(A)* total scan photo diode array (PDA) chromatogram. Chromatogram of the ion at (B) *m/z* 403 in positive ionization mode (tryptoquivaline F), (C) *m/z* 444 in positive ionization mode (fumiquinazoline C), (D) *m*/*z* 345 in positive ionization mode (trypacidin), (E) *m*/*z* 345 in negative ionization mode (monomethylsulochrin), (F) *m*/*z* 283 in negative ionization mode (questin).

Consequently, extracts of conidia from the other wild strain (NRRL 35693) were also analyzed by LC-MS (Figure 5). All the five metabolites, trypacidin, monomethylsulochrin, questin, tryptoquivaline F and fumiquinazoline C were detected in the conidial extracts. These 5 compounds, moreover, were also detected by LC-MS analysis in all of the 17 *A. fumigatus* culture extracts mentioned in Table S1.

### Identification of the toxic metabolites produced by *A. fumigatus*

**Figure 6:**
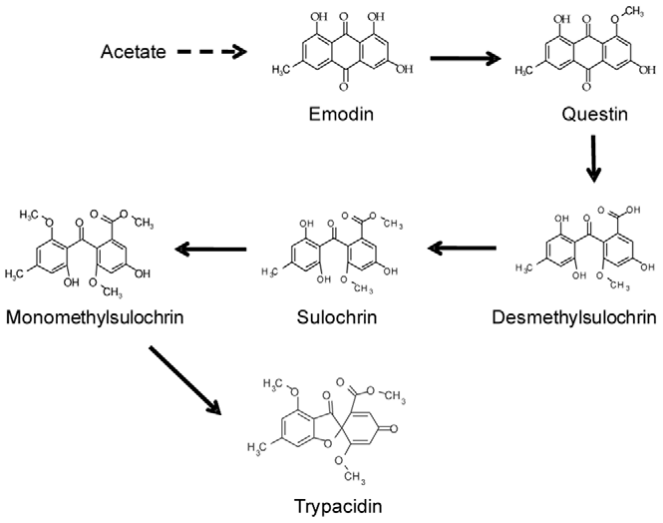
Outline biosynthesic pathway for trypacidin.

Fractions obtained from chromatoflash and containing the 5 main toxins borne by conidia, were tested for their cytotoxicity to A549 cells using the MTT assay. The composition of each fraction regarding other secondary metabolites produced by *Aspergillus fumigatus* was determined by LC-MS analysis (Table 1). In addition to the airborne metabolites, fumagillin, verruculogen, helvolic acid, fumigaclavine A were also detected in those fractions. The most toxic fractions (F14, F16, F18) contained all metabolites. It is noteworthy, however, that the elution pattern of trypacidin, fumagillin and fumigaclavine A shared a profile similar to that of the toxicity of the fractions. The significance of the two latter compounds, however, could be discounted since 100 µM fumagillin or fumigaclavine A did not trigger any significant loss of viability (106.0±5.6% and 107.2±6.0% cell viability respectively). Since F16 fraction displayed the highest toxic activity compared to the other fractions whatever the tested dilution (Figure S2), it was chosen to be fractionated on reverse-phase-HPLC (Figure S3). The measurement of the toxicity and the metabolite content of the resulting sub-fractions showed that the fractions containing tryptoquivaline F and fumiquinazoline C had very low toxicity (Table 2). The most toxic fraction F6, conversely matched the peaks of trypacidin, monomethylsulochrin and questin but also fumagillin. When tested alone, however, fumagillin and verruculogen showed no detectable toxicity even at the high concentration of 100 µM thereby confirming that the toxicity of F6 and F7 was due to the presence of trypacidin, monomethylsulochrin and questin. It is noteworthy that these three metabolites are components of the pathway leading to trypacidin synthesis (Figure 6). The toxic activities induced by fractions F8, F9 and F10 were correlated to the presence of an unknown compound which does not absorb from 200 to 600 nm but displayed a base peak at *m*/*z* 453 in LC-MS positive mode.

**Table 1:** Comparison of LC-MS analysis of the fractions obtained from flash chromatography and their effect on the A549 cell viability.

	Fraction							
	control	F12	F14	F16	F18	F19	F20	F30
**Metabolites**	Relative LC-MS peak intensity							
Fumagillin		58.2	100	60.2	41.8	nd	nd	nd
Trypacidin		27.2	100	100	81.7	24.5	70.0	nd
Fumigaclavine A		nd	46.3	100	47.2	26.0	nd	nd
Helvolic acid		nd	56.5	83.1	100	77.2	nd	nd
Monomethylsulochrin		nd	11.1	89.2	100	92.3	98.5	nd
Questin		1.3	5.2	57.4	100	81.8	100	1.7
Verruculogen		12.6	53.6	53.6	88.9	97.5	100	11.5
Fumiquinazoline C		nd	18.2	49.3	48.5	61.3	100	nd
Tryptoquivaline F		9.8	5.7	23.8	29.5	44.0	100	43.5
***cell viability (%)***	*95.2*	*34.2*	*2.1*	*2.8*	*0.2*	*34.3*	*22.6*	*88.6*

Fractionation of bulk culture extracts using flash chromatography. To measure their toxicity, each 5 ml fraction was dried and solubilised in 500 µl in PBS-DMSO 40% and then diluted 10 fold in PBS-DMSO 12%. The final concentration of DMSO in the assay medium was 1.2%. The cells were exposed for 24 h before measuring the cell viability as described in the Materials and Methods. The effect of 1.2% DMSO was used as negative control. For each metabolite, the figures are arbitrary units indicating its elution profile with 100 corresponding to the maximum normalized level measured by the LC-MS sensor. nd means not detectable.

**Table 2:** Comparison of LC-MS analysis of the F16 sub-fractions obtained from the RP-HPLC and their effect on the A549 cell viability.

	Fraction								
	control	F3	F4	F5	F6	F7	F8	F9	F10
**Metabolites**	Relative LC-MS peak intensity								
Tryptoquivaline F		nd	42.0	100	6.7	nd	nd	nd	nd
Fumiquinazoline C		nd	31.3	100	8.3	4.2	nd	nd	nd
Fumagillin			nd	97.5	100	93.1	nd	nd	nd
Trypacidin				nd	100	8.8	3.0	nd	nd
Monomethylsulochrin				nd	100	40.9	2.3	nd	nd
Questin				nd	100	54.7	1.6	nd	nd
Verruculogen				nd	nd	100	58.9	nd	nd
Helvolic acid				nd	nd	nd	100	nd	nd
Fumitremorgin B				nd	nd	nd	100	nd	nd
Emodin				nd	nd	nd	nd	nd	nd
Fumigaclavine A				nd	nd	nd	nd	nd	nd
Unknown (*m*/*z* 453)*				nd	nd	nd	60.3	100	48.1
***cell viability (%)***	*92.5*	*80.7*	*79.0*	*77.2*	*35.5*	*48.7*	*47.0*	*47.1*	*70.4*

Sub-fractionation of F16 fraction using RP-HPLC. F3 to F7 were each collected for 2 min, F8 for 5 min, F9 for 10 min and F10 for 6 min. Each fraction was evaporated and the dried material was dissolved in 50 µl PBS-DMSO 12%. The final concentration of DMSO in the assay medium was 1.2%. The cell viability was measured by the MTT assay described in the Materials and Methods after exposing A549 cells to the metabolites for 48 h. The effect of 1.2% DMSO was used as negative control. For each metabolite, the figures are arbitrary units indicating its elution profile, 100 corresponding to the maximum normalized level measured by the LC-MS sensor. nd means not detectable.

*m/*z* in positive mode electrospray ionization.

### Toxicity of trypacidin and its intermediate metabolites

**Figure 7:**
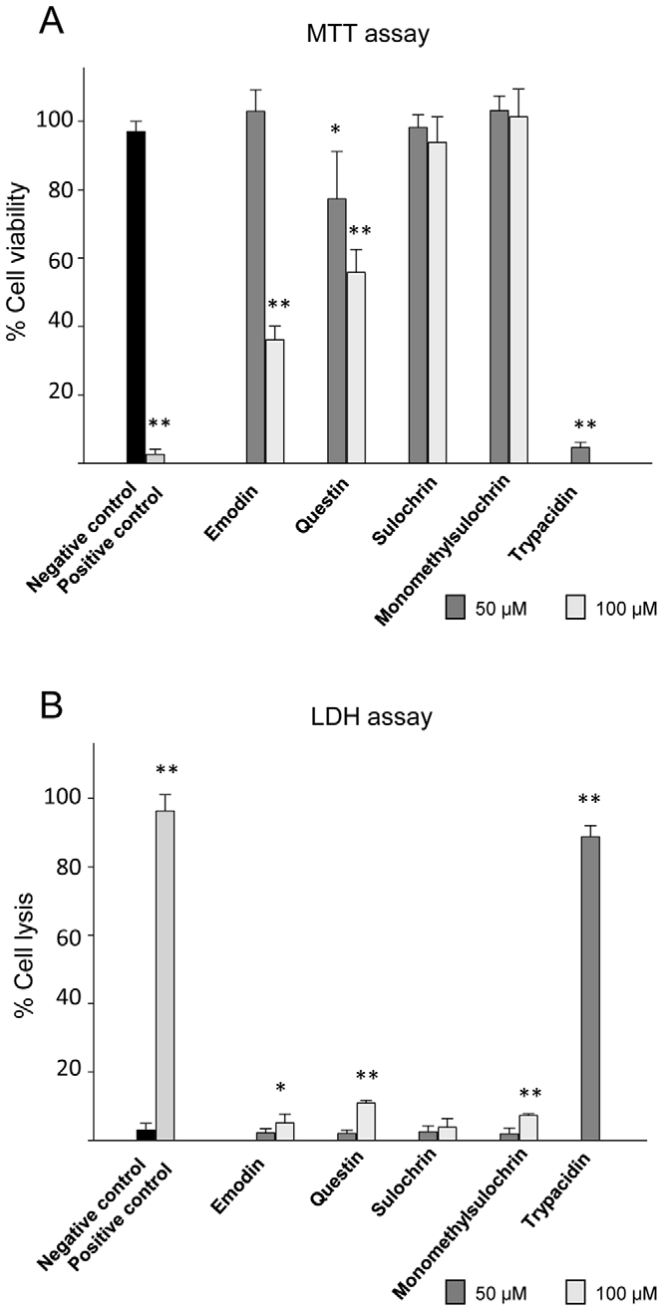
Toxicity effect of the trypacidin pathway metabolites on A549 cells. Cell viability and cell lysis were measured using respectively MTT and LDH assay. The cells were exposed for 24 h in culture conditions to each metabolite before measuring cell viability and cell lysis as described in the Materials and Methods. The graph shows the mean values of three independent experiments, excepted for trypacidin for which five experiments were carried out. Negative and positive control corresponds respectively to 1% DMSO and gliotoxin (10 µM for MTT assay and 50 µM for LDH assay). Trypacidin was tested at 50 µM and the other metabolites at 50 and 100 µM. Significant difference from the negative control was analysed with the t-test (_*_ for p<0.05; _**_ for p<0.001).

These three metabolites were purified by RP-HPLC and their chemical structures verified by NMR. Their toxicity was compared with emodin and sulochrin, two other commercially available intermediate metabolites of the trypacidin pathway, using the MTT and LDH assays (Figure 7). The results show that 50 µM trypacidin reduced cell viability by nearly 100% and triggered 85% cell lysis while the other metabolites showed only slight or no toxicity at this concentration. Even at 100 µM, sulochrin and monomethylsulochrin still showed no effects in either assay whereas both emodin and questin had a significant (p<0.001) but minor effect on the cell viability (64±4% and 44±7% inhibition, respectively) and little effect on cell lysis. These results indicated that trypacidin was much more toxic than its precursors and is the most toxic compound among the five tested metabolites.

### Dose and time dependent effect of trypacidin on A549 cells

The dose-effect study of trypacidin on cell viability and cell lysis was determined on A549 cells after 24 h exposure in culture. The results show a dose-dependence at concentrations ranging from 0.5 to 50 µM (Figure S4). Trypacidin gave the same IC_50_ for cell viability and cell lysis, respectively 7.4±2.0 µM (n = 5) and 7.4±2.4 µM (n = 5) indicating that at the same concentration the loss of the cell viability observed after 24 h exposure was effecting cell lysis.

When the cells were exposed to 50 µM trypacidin for only 1 or 2 h, the cell viability measured after 24 h of culture was the same as that measured when the cells were exposed to the same concentration of trypacidin for 24 h (Table 3). These results show that trypacidin rapidly induced an irreversible process leading to the cell death within 24 h.

**Table 3:** Inhibition of A549 cell viability by trypacidin.

	Trypacidin µM	
	**10**	**50**

Exposure (h)	% of cell viability	
1	**43±3**	**2±2**
2	**41±2**	**3±2**
24	**38±5**	**1±1**

A549 cells were exposed for 1 h and 2 h with 10 µM and 50 µM trypacidin, then the medium containing trypacidin was replaced by fresh medium and cell viability was measured 24 h later using MTT assay. Cells were also exposed to trypacidin for 24 h before the MTT assay.

### Production of ROS and RNS following trypacidin exposure

**Figure 8:**
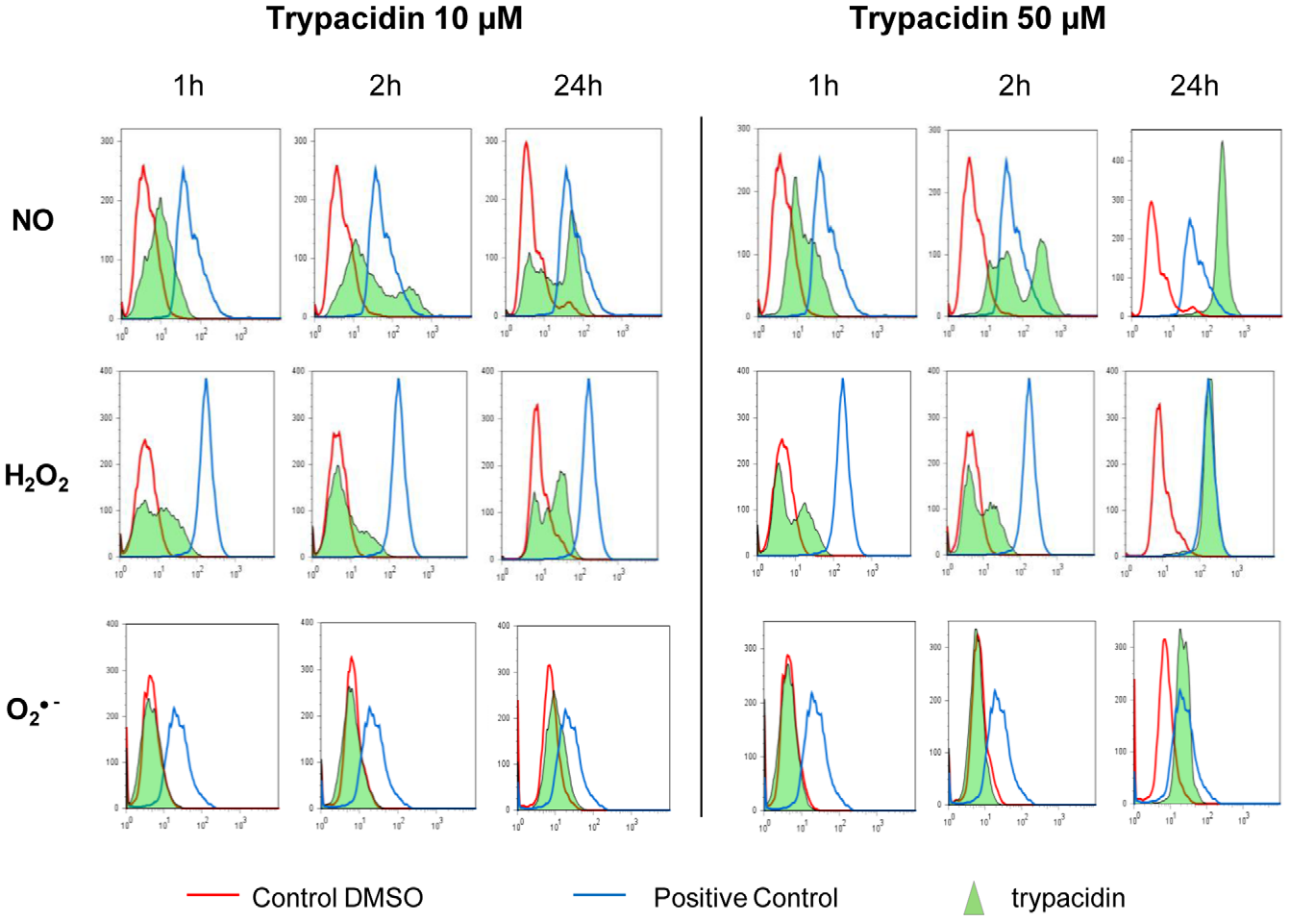
Flow cytometry analysis of ROS and RNS production. A549 cells were seeded at 3×10^4^ cells/cm^2^ and cultured for 24 h. Then, cells were exposed to trypacidin (green area) 10 µM and 50 µM or DMSO 1% (red line) for 1 h, 2 h and 24 h. H_2_O_2_ production was assessed using H2-DCF-DA, O^2•−^ using DHE, NO using DAF-FM. Menadione was used as control for H2-DCF-DA and DHE, SNAP for DAF-FM (blue line). Peaks are representative of three independent experiments.

To determine whether A549 cells produced ROS and RNS in response to trypacidin exposure, we used fluorescent probes to measure the formation of O_2_^•−^, H_2_O_2_ and NO following short (1 h, 2 h) and long (24 h) exposures to 10 µM and 50 µM of the toxin (Figure 8). Production of H_2_O_2_ and NO was observed as soon as one hour exposure while, in contrast, no detectable formation of O_2_^•−^ was observed within the same time period.

**Figure 9:**
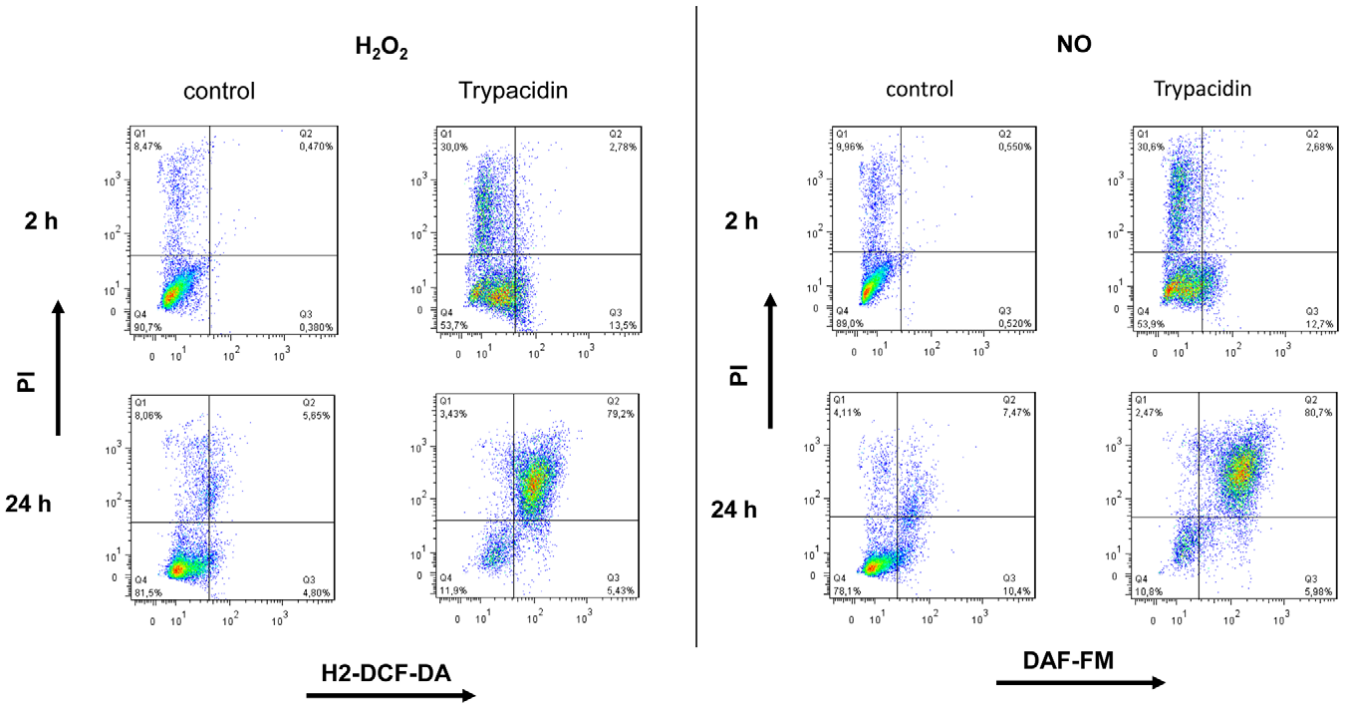
Flow cytometry analysis of H_2_O_2_ and NO related to PI staining. A549 cells were seeded at 3×10^4^ cells/cm^2^ and cultured for 24 h. Then, cells were exposed to trypacidin 50 µM or DMSO 1% for 2 h and 24 h. H_2_O_2_ and NO production were measured using respectively H2-DCF-DA, and DAF-FM. IP was used to stain dead cells. Dot-blots are representative of three independent experiments.

After 24 h exposure, 10 µM trypacidin induced H_2_O_2_ and NO production in about 50% of the cell population while 50 µM trypacidin induced production of H_2_O_2_, NO but also O_2_^•−^ in the whole cell population. In that latter condition, most of the NO and H_2_O_2_ producing cells were dead (80% and 70% respectively) as shown by propidium iodide staining (Figure 9). It can be concluded that the first hours of trypacidin exposure triggered an early cell death of a minor part of the cell population and that the later cell death appeared after oxidative injury.

Taken together, those results suggested that trypacidin induced an oxidative stress in the cells which was strongly implicated in the death process.

### Effect of trypacidin on the cell cycle and apoptosis

**Figure 10:**
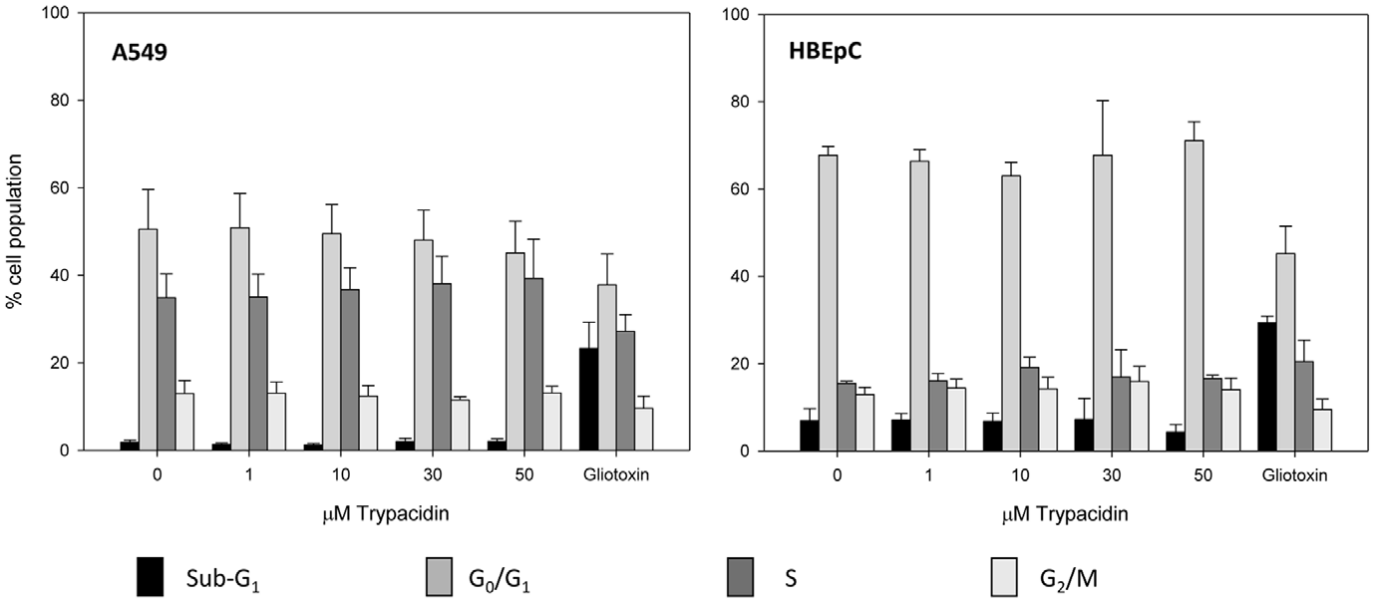
Flow cytometry analysis of the cell cycle. A549 and HBEpC were exposed to 1, 10, 30 and 50 µM trypacidin for 24 h in culture conditions. In the negative control (0 µM trypacidin), cells were exposed to 1% DMSO. Gliotoxin 5 µM was used as positive control to induce sub-G1 particles. The cells were then harvested and analysed for cell cycle as described in the Materials and Methods. The figure shows mean values for 3 independent experiments.

**Figure 11:**
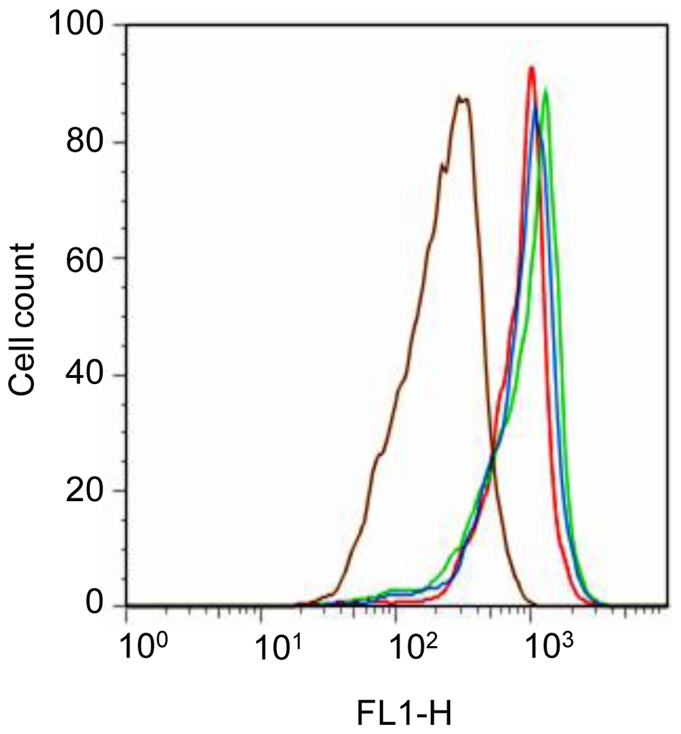
Assessment of ΔΨm. A549 cells were exposed 2 h or 24 h to 10 µM (green line) and 50 µM (blue line) trypacidin or with solely 1% DMSO as negative control (red line). After treatment, cells were stained with DiOC_6_ for 20 min. at 37°C. Cells incubated with 40 µM FCCP were used as positive control (brown line). The figure is representative of 3 independent experiments.

In order to determine if trypacidin was able to arrest the cell cycle or to induce apoptosis, flow cytometry was used to examine any modification of the G0/G1, S, G2/M phase ratio or appearance of a sub-G1 fraction (Figure 10), which is typical of apoptotic bodies. The experiment was performed with A549 alveolar lung cells and with bronchial epithelial cells (HBEpC), two types of respiratory tract cells. Indeed, since A549 is a cancer cell line, which could result in impairment of the apoptotic pathway, so it was important to obtain results with primary lung cells. The toxicity of trypacidin against HBEpC cells was previously determined with the MTT assay under the same conditions used for the A549 line. The IC_50_ obtained (9.2±2.0 µM; n = 6) was not significantly different from the IC_50_ obtained with the A549 cells. Figure 10 shows that at concentrations from 1 µM to 50 µM, trypacidin did not alter the cell cycle and did not induce sub-G1 particles in either cell type suggesting that the toxin did not trigger the formation of apoptotic bodies. This latter result was confirmed by the measurement of the mitochondrial membrane potential (ΔΨ*m*) in the A549 cells (Figure 11). A reduction in the ΔΨ*m* is an early event in apoptosis caused by the leakage of protons from the intermembrane space of the mitochondria due to the formation of pores in the mitochondrial membrane [28]. After 2 h or 24 h exposure to 10 or 50 µM trypacidin, the A549 cells did not show any loss of mitochondrial potential.

These results showed that trypacidin neither arrested the cell cycle nor induced apoptosis in contrast to the effects of 5 µM gliotoxin which induced 23.3±6.0% and 29.4±1.5% sub-G1 particles in A549 and HBEpC cell populations respectively.

## Discussion

Since the identification of trypacidin and the demonstration of its anti-protozoal properties against *Trypanosoma cruzi* and *Toxoplasma gondii* in 1963 [22], no study had been performed regarding its toxicity to mammalian cells. This is the first time that the toxicity of this secondary metabolite has been demonstrated, which means it can be considered to be a mycotoxin.

A large number of secondary metabolites are potentially produced by *Aspergillus fumigatus*, depending on the growth conditions of the fungus. Only a limited number are airborne, however, carried by the spores. We could identify metabolites that were produced when *A. fumigatus* sporulated but were not detected in the conidiogenesis deficient Δ*brlA* strain culture. The absence of fumigaclavines production in Δ*brlA* strain is consistent with de BrlAp dependence of genes involved in biosynthesis of these secondary metabolites as shown previously in a transcriptome study [29]. Likewise, the absence of fumiquinazoline C production in Δ*brlA* strain is consistent with the down-regulation of two genes (Afu6g12050 and Afu6g12070) essential for its synthesis [30], [31]. By contrast, the fumitremorgens cluster [32] was reported to be suppressed by BrlAp, explaining the strong production of fumitremorgins and verruculogen in Δ*brlA* strain. Since the genes cluster involved in trypacidin biosynthesis has not been yet characterized it was not possible to compare our metabolome data with the transcriptome data previously published [29].

Fischer et al. reported that trypacidin and a tryptoquivaline were both found in *A. fumigatus* spore extracts and in bioaerosols from compost [21]. Doubts still remained, however, about the effective production of tryptoquivalines by *A. fumigatus*. A previous report [33] had postulated that fumiquinazolines had been misidentified as tryptoquivalines in Fischer's study since tryptoquivalines and fumiquinazolines share similar UV spectra [15]. This was also seen in other previous studies [34], [35], [36]. Tryptoquivaline J was, however, isolated recently from an *A. fumigatus* strain by Afiyatullov [37]. The current results confirm the effective synthesis of tryptoquivalines by this species. They also confirm that trypacidin and tryptoquivaline F/J are borne by the spores and that 3 other metabolites, monomethylsulochrin, questin and fumiquinazoline C are also present in the spores of *A. fumigatus*. The toxicity of those 5 compounds on airway tissues, however, had not been studied until now.

Trypacidin was found to be the most toxic metabolite borne by the spores in the current study. The fungal precursor metabolites of trypacidin biosynthesis had low toxicity compared with trypacidin. Sulochrin and monomethylsulochrin did not cause cell lysis or reduce cell viability whereas the two anthraquinone derivatives, emodin and questin, reduced only cell viability at respectively 100 µM and 50 µM. These results are partially in agreement with a previous report showing that sulochrin and questin did not trigger any toxic effects on A549 cells at 150 µM using the same MTT assay [38]. It should be noted, however, that these authors used a different process of purification to obtain questin. This could mean that other compounds present in the questin preparation could overestimate the calculated 150 µM concentration used in the MTT assay.

The toxicity of trypacidin was assayed with A549 alveolar lung cells and human bronchial epithelial cells which are representative of the upper and lower airway respiratory tract, respectively. Toxicity was dose-dependent with both cell types with a 50% maximum effect around 7 µM. Trypacidin initiated a process of cell death within the first hour of exposure leading to cell necrosis within 24 h as shown by the release of the cytosolic enzyme LDH.

Other mycotoxins such as gliotoxin, aflatoxin B1 produced by the *Aspergillus* genus and deoxynivalenol, a trichothecene mycotoxin produced by several *Fusarium* species, were previously reported to trigger ROS formation within 1 h of exposure in different types of cells. These included a porcine renal proximal tubular cell line [39], hepatic stellate cells [40], hepatocytes [41], hepatoma cells HepG2 [42], and colon HT29 cells [43].

ROS and RNS are known to oxidize cell constituents such as DNA, proteins and lipids, leading to cell damage or death.

It was thus of interest to assess whether trypacidin would induce the formation of ROS and RNS in A549 cells. Our results show clearly that trypacidin induces hydrogen peroxide (H_2_O_2_) and nitric oxide (NO) within the first hour of exposure and that their accumulation after 24 h exposure is correlated with cell lysis as shown by the LDH release. It can be noted that O_2_^•−^ accumulated in A549 cells only when levels of NO and H_2_O_2_ dramatically increased. H_2_O_2_ is produced from O_2_^•−^ through superoxide dismutase (SOD) activity. The O_2_^•−^ accumulation could be due to the inhibition of SOD activity by an exceeding level of H_2_O_2_.

In the current study, trypacidin did not induce either an arrest of the cell cycle or apoptotic bodies in lung alveolar and bronchial epithelial cells. It was also observed that trypacidin did not induce a collapse of the mitochondrial transmembrane potential of A549 cells. This confirms that the apoptosis machinery was not initiated by the trypacidin toxicity.

The unchanged mitochondrial transmembrane potential, however, suggests an inhibition of the electron transport chain without pore formation. These results are consistent with a previous study showing that culture filtrates from *A. fumigatus* did not cause membrane blebbing and apoptotic bodies in A549 cells and that the cells died from necrosis [44]. It has been shown, furthermore, that conidia from *A. fumigatus* isolated from humans, birds and the environment were able to inhibit apoptosis induced in A549 cells and 16HBE bronchial epithelial cells [45].

The current study shows that conidia from *A. fumigatus* mainly carry 5 secondary metabolites but only trypacidin is highly toxic to lung cells. However, the analysis of eight *Aspergillus* genomes, and particularly that of *A. fumigatus* genome [12], [13], revealed a number of PKS and NRPS genes much greater than the number predicted known secondary metabolites. Given the existence of genes clusters silent under standard laboratory conditions [46], [47], our results do not exclude the possibility of cryptic spore-born products not activated in our culture conditions, but induced in field conditions.

Accumulation of spores in cavities of the alveoli (about 4 nl) could lead to pneumocytes being exposed to a toxic level of trypacidin. *In vivo* studies have confirmed that other airborne spore mycotoxins can induce pulmonary inflammation. Low doses (4×10^−5^ mole/kg lung wt) of atranone C, brevianamide, cladosporin, mycophenolic acid, neoechinulin and sterigmatocystin induced pulmonary inflammation when applied intratracheally in mice [48]. It was also shown that instillation into mice of a low number of spores from *Stachybotrys chartarum* (30 spores/g body weight) is cytotoxic for lung cells with LDH being released in the bronchoalveolar lavage fluid [49].

Additional work has to be performed in order to know whether trypacidin could similarly induce lung cell necrosis *in vivo*.

## Supporting Information

Figure S1 **NMR spectrum data of questin, monomethylsulochrin, trypacidin, fumiquinazoline C, tryptoquivaline F.** ^1^H and two-dimensional nuclear magnetic resonance (2D-NMR) spectra were generated using a Bruker Avance DRX-600 spectrometer operating at 600.13 MHz in CDCl_3_ solution as described in Materials and Methods.
(DOC)

Figure S2 **Toxicity of fractions issued from flash chromatography.** The toxic effect of fractions F12, F14, F16, F18, F19, F20 were measured at four different dilutions in PBS-DMSO 12%: 1/10, 1/50, 1/100, 1/500. Ten µl of each fraction were added to wells containing 100 µl of culture medium. The cells were exposed for 24 h before measuring the cell viability using the MTT assay as described in the Materials and Methods.
(PDF)

Figure S3 **Sub-fractionation of F16 fraction using RP-HPLC.** F16 fraction was evaporated, dissolved in methanol and fractionated using RP-HPLC. Ten fractions were collected as follows: F1 (1–10 min), F2 (10–15 min), F3 (15–17 min), F4 (17–19 min), F5 (19–21 min), F6 (21–23 min), F7 (23–25 min), F8 (25–30 min), F9 (30–40 min) and F10 (40–46 min). Each subfraction was analysed by HPLC-DAD and LC-MS. A) HPLC chromatogram at 270 nm of fraction F16. B) PDA total scan [200–600 nm] chromatogram of subfractions 5–10.
(PDF)

Figure S4 **Dose dependent effect of trypacidin on cell viability and cell lysis.** 1×10^4^ A549 cells were cultured for 24 h in 96-well plates and then exposed to trypacidin for 24 h. Cell viability and cell lysis were measured as described in Materials and Methods using the MTT and LDH assays. The figure is representative of 5 independent experiments. The toxin concentration triggering 50% toxic effect (IC_50_) was calculated using the software SigmaPlot.
(PDF)

Table S1 ***Aspergillus fumigatus*** **strains studied.**
(DOC)
