# Supplementary material for: Trypacidin, a Spore-Borne Toxin from Aspergillus fumigatus, Is Cytotoxic to Lung Cells
Source: PLoS One. 2012 Feb 3;7(2):e29906. doi: 10.1371/journal.pone.0029906 (PMC3272003; doi:10.1371/journal.pone.0029906)
Supplement: Figure S1 — NMR spectrum data of questin, monomethylsulochrin, trypacidin, fumiquinazoline C, tryptoquivaline F. 1H and two-dimensional nuclear magnetic resonance (2D-NMR) spectra were generated using a Bruker Avance DRX-600 spectrometer operating at 600.13 MHz in CDCl3 solution as described in Materials and Methods. (DOC) [file pone.0029906.s001.doc]

SUPPLEMENTAL MATERIAL S1 : NMR spectrum data

Questin :70 µg.

^1^H NMR (600 MHz, CDCl_3_) δ ppm : 13.1 (1H,s,OH-1); 7.58 (1H, d, J=1.5 Hz, H-4); 7.36 (1H, d, J=2 Hz, H-5); 7.08 (1H, d, J=1.5 Hz, H-2); 6.80 (1H, d, J=2 Hz, H-7); 4.04 (3H, s, OCH_3_-8); 2.44 (3H, s, CH_3_-3).

^13^C NMR (150 MHz, CDCl_3_) δ ppm: 182.7 (C-10); 163.3 (C-6); 161.8 (C-8); 146.9 (C-3); 124.6 (C-2); 120.2 (C-4); 115.0 (C-8a); 114.6 (C-9a); 106.9 (C-5); 104.4 (C-7); 56.6 (OCH_3_-8); 21.8 (CH_3_-3).

Monomethylsulochrin: 150 µg

^1^H NMR (600 MHz, CDCl_3_) δ ppm : 7.03 (1H, d, J=2.2 Hz, H-5); 6.63 (1H, d, J=2.2 Hz, H-3); 6.46 (1H, brs, H-5’); 6.07 (1H, brs, H-3’); 3.71 (3H, s, H-9); 3.69 (3H, s, H-8); 3.37 (3H, s, H-7’); 2.29 (3H, s, H-8’).

^13^C NMR (150 MHz, CDCl_3_) δ ppm : 165.8 (C-7); 160.8 (C-2’); 157.2 (C-2); 155.7 (C-4); 148.1 (C-4’); 128.2 (C-1); 110.8 (C-5’); 110.2 (C-1’); 107.4 (C-5); 102.9 (C-3); 102.7 (C-3’); 56.0 (C-9); 55.4 (C-7’); 52.1 (C-8); 22.4 (C-8’).

Trypacidin: 150 µg

^1^H NMR (600 MHz, CDCl_3_) δ ppm : 7.03 (1H, d, J=2.2 Hz, H-5); 6.63 (1H, d, J=2.2 Hz, H-3); 6.46 (1H, brs, H-5’); 6.07 (1H, brs, H-3’); 3.71 (3H, s, H-9); 3.69 (3H, s, H-8); 3.37 (3H, s, H-7’); 2.29 (3H, s, H-8’).

^13^C NMR (150 MHz, CDCl_3_) δ ppm : 174.1 (C-1); 169.0 (C-13); 163.3 (C-17); 158.2 (C-5); 151.8 (C-3); 136.9 (C-10); 108.1 (C-6); 105.2 (C-2); 104.9 (C-4); 103.6 (C-12); 83.9 (C-8); 56.4 (C-16); 55.8 (C-15); 52.6 (C-18); 22.9 (C-14).

Fumiquinazoline C : 200 µg

^1^H NMR (600 MHz, CDCl_3_) δ ppm : 8.36 (1H, dd, J=7.8 and 1.5 Hz, H-10); 7.86 (1H, t, J=7.8 Hz, H-8) ; 7.80 (1H, dd, J=7.8 and 1.5 Hz, H-7) ; 7.63 (1H, t, J=7.8 Hz, H-9) ; 7.46 (1H, d, J=7.7 Hz, H-24) ; 7.34 (1H, d, J=7.7 Hz, H-27) ; 7.32 (1H, t, J=7.7 Hz, H-25) ; 7.20 (1H, t, J=7.7 Hz, H-26) ; 6.61 (1H, brs, H-2); 5.74 (1H, d, J=7.4 Hz, H-14); 5.34 (1H, d, J=7.3 Hz, H-18); 3.70 (1H, qui, J=7.6 Hz, H-20); 2.98 (1H, dd, J=15.3 and 7.6 Hz, H-15A); 2.12 (1H, d, J=15 Hz, H-15B); 2.06 (3H, s, H-16) ; 1.07 (3H,d, J=6.8 Hz, H-29) ; 1.03 (1H, t, J=7.7 Hz, H-19).

Tryptoquivaline F or J : 200 µg

^1^H NMR (600 MHz, CDCl_3_) δ ppm : 8.29 (1H, d, J=7.9, H-20); 7.90 (1H, s, H-26) ; 7.77 (1H, td, J=7.0 and 1.0 Hz, H-22) ; 7.72 (1H, d, J=8.1 Hz, H-23) ; 7.66 (1H, d, J=8 Hz, H-5) ; 7.51 (1H, t, J=7.1 Hz, H-21) ; 7.47 (1H, t, J=7.6 Hz, H-6) ; 7.39 (1H, d, J=7.6 Hz, H-8) ; 7.25 (1H, t, J=7.6 Hz, H-7); 5.57 (1H, s, H-2); 4.50 (1H, q, J=6.9 Hz, H-15); 1.80 (3H, d, J=6.9 Hz, H-31).
