# Supplementary material for: Trypacidin, a Spore-Borne Toxin from Aspergillus fumigatus, Is Cytotoxic to Lung Cells
Source: PLoS One. 2012 Feb 3;7(2):e29906. doi: 10.1371/journal.pone.0029906 (PMC3272003; doi:10.1371/journal.pone.0029906)
Supplement: Figure S2 — Toxicity of fractions issued from flash chromatography. The toxic effect of fractions F12, F14, F16, F18, F19, F20 were measured at four different dilutions in PBS-DMSO 12%: 1/10, 1/50, 1/100, 1/500. Ten µl of each fraction were added to wells containing 100 µl of culture medium. The cells were exposed for 24 h before measuring the cell viability using the MTT assay as described in the Materials and Methods. (PDF) [file pone.0029906.s002.pdf]

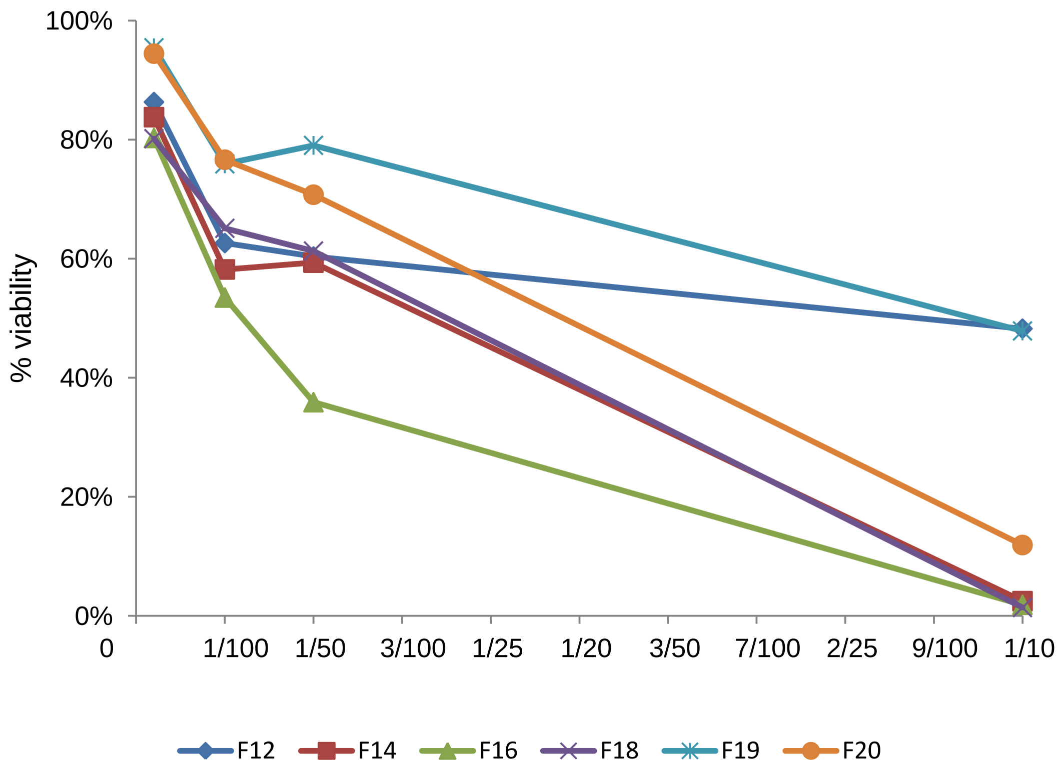

Figure S2. Toxicity of fractions issued from flash chromatography. The toxic effect of fractions F12, F14, F16, F18, F19, F20 were measured at four different dilutions in PBS-DMSO 12%: 1/10, 1/50, 1/100, 1/500. Ten µl were added to each well containing 100 µl culture medium. The cells were exposed for 24 hours before measuring the cell viability as described in the Materials and Methods.
