# Supplementary material for: Trypacidin, a Spore-Borne Toxin from Aspergillus fumigatus, Is Cytotoxic to Lung Cells
Source: PLoS One. 2012 Feb 3;7(2):e29906. doi: 10.1371/journal.pone.0029906 (PMC3272003; doi:10.1371/journal.pone.0029906)
Supplement: Figure S3 — Sub-fractionation of F16 fraction using RP-HPLC. F16 fraction was evaporated, dissolved in methanol and fractionated using RP-HPLC. Ten fractions were collected as follows: F1 (1–10 min), F2 (10–15 min), F3 (15–17 min), F4 (17–19 min), F5 (19–21 min), F6 (21–23 min), F7 (23–25 min), F8 (25–30 min), F9 (30–40 min) and F10 (40–46 min). Each subfraction was analysed by HPLC-DAD and LC-MS. A) HPLC chromatogram at 270 nm of fraction F16. B) PDA total scan [200–600 nm] chromatogram of subfractions 5–10. (PDF) [file pone.0029906.s003.pdf]

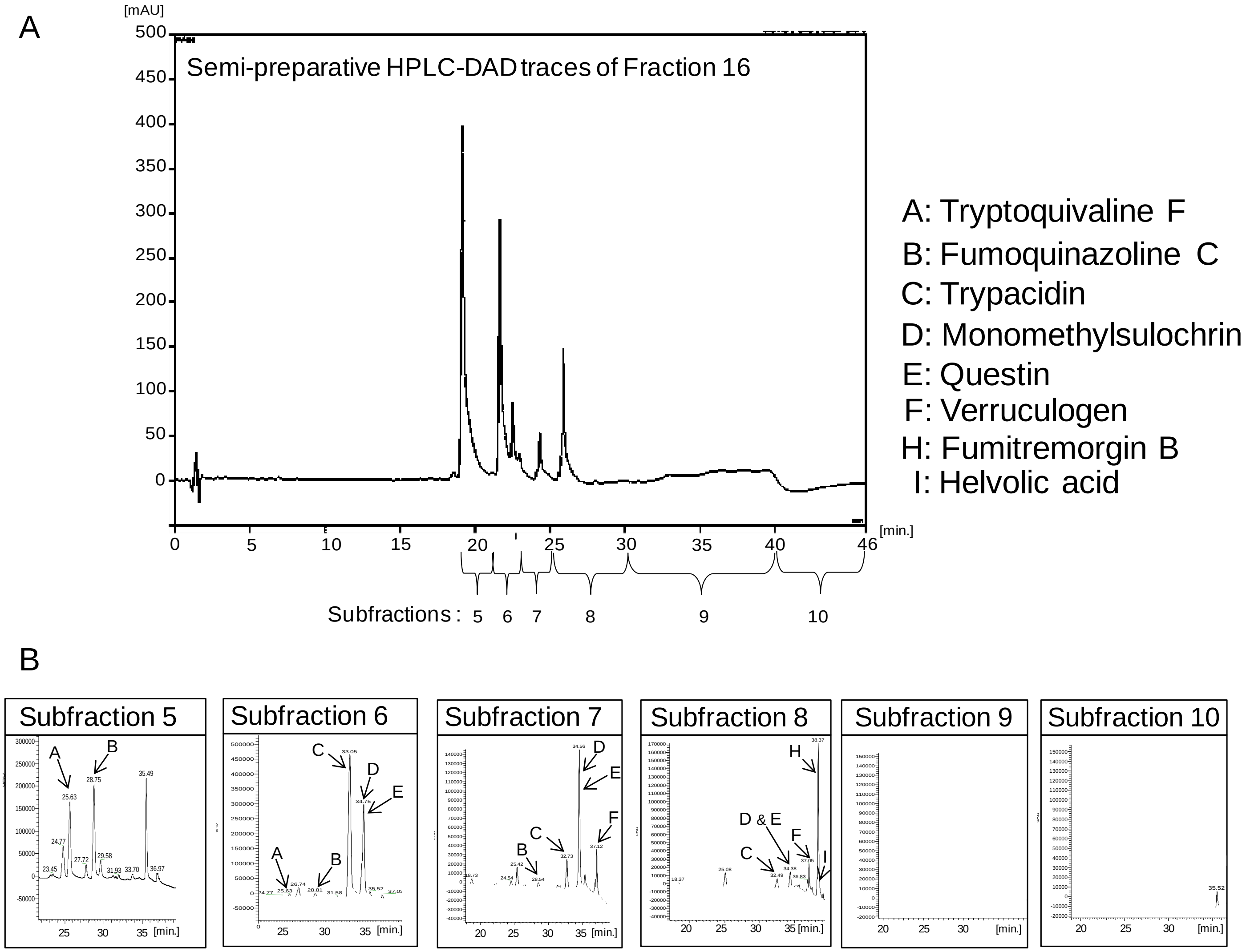

Figure S3. Subfractionation of F16 fraction using RP-HPLC. F16 fraction was evaporated, dissolved in methanol and fractionated using RP-HPLC. Ten fractions were collected as follows: F1 (1-10 min), F2 (10-15 min), F3 (15-17 min), F4 (17-19 min), F5 (19-21 min), F6 (21-23 min), F7 (23-25 min), F8 (25-30 min), F9 (30-40 min), F10 (40-46 min). Each subfraction was analysed by HPLC-DAD and LC-MS. A) HPLC chromatogram at 270 nm of fraction F16. B) PDA total scan [200-600 nm] chromatogram of subfractions 5-10.
