# Supplementary material for: Trypacidin, a Spore-Borne Toxin from Aspergillus fumigatus, Is Cytotoxic to Lung Cells
Source: PLoS One. 2012 Feb 3;7(2):e29906. doi: 10.1371/journal.pone.0029906 (PMC3272003; doi:10.1371/journal.pone.0029906)
Supplement: Figure S4 — Dose dependent effect of trypacidin on cell viability and cell lysis. 1×10^4^ A549 cells were cultured for 24 h in 96-well plates and then exposed to trypacidin for 24 h. Cell viability and cell lysis were measured as described in Materials and Methods using the MTT and LDH assays. The figure is representative of 5 independent experiments. The toxin concentration triggering 50% toxic effect (IC50) was calculated using the software SigmaPlot. (PDF) [file pone.0029906.s004.pdf]

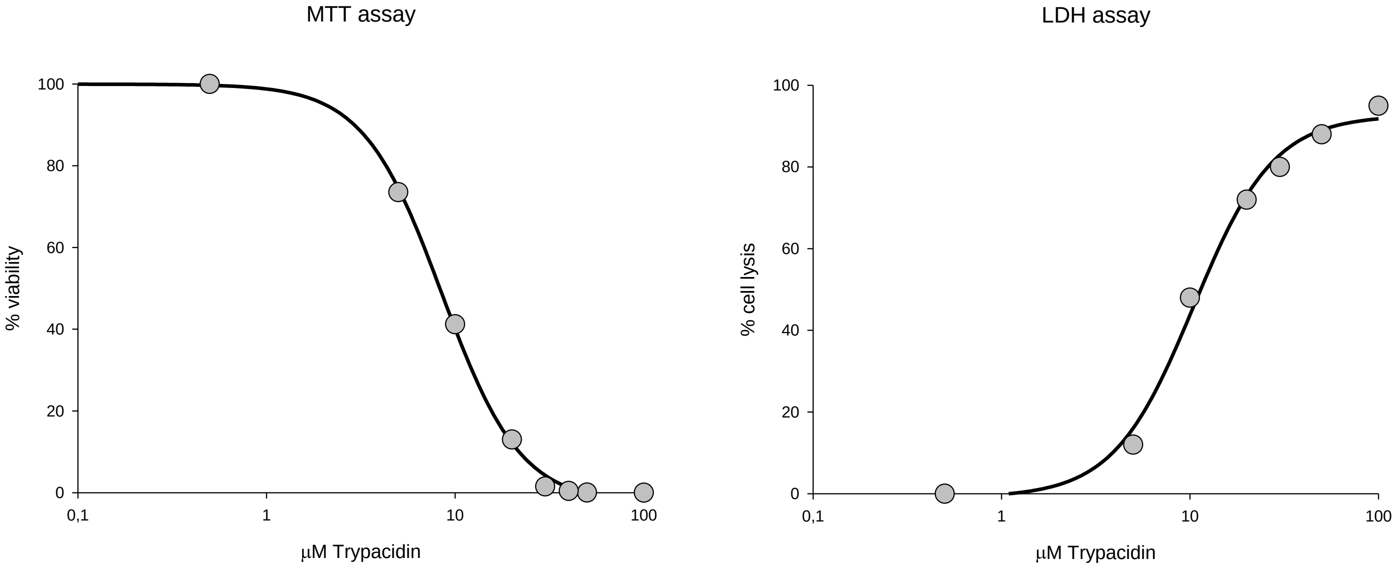

Figure S4. Dose dependent effect of trypacidin on cell viability and cell lysis. 1×10^4 A549 cells were cultured for 24 hours in 96-well plates and then exposed to trypacidin for 24 hours. Cell viability and cell lysis were measured as described in Materials and Methods using the MTT and LDH assays. The toxin concentration triggering 50% toxic effect (IC50) was calculated using the software SigmaPlot.
