# Supplementary material for: Trypacidin, a Spore-Borne Toxin from Aspergillus fumigatus, Is Cytotoxic to Lung Cells
Source: PLoS One. 2012 Feb 3;7(2):e29906. doi: 10.1371/journal.pone.0029906 (PMC3272003; doi:10.1371/journal.pone.0029906)
Supplement: Table S1 — Aspergillus fumigatus strains studied. (DOC) [file pone.0029906.s005.doc]

Table S1: *Aspergillus fumigatus* strains studied.

| Strain | Source |
| --- | --- |
| NCPT 13 (NRRL 35693) | barley grains |
| Mar 1154.2 | patient |
| Gem 9683.1 | patient |
| Loh 1035.3 | patient |
| HD 94 | hospital environment |
| LOU 150.53 | patient |
| HD 230 | hospital environment |
| Mar 6389.3 | patient |
| LOU 3065.3 | patient |
| A 246 | patient |
| LAR 3132.3 | patient |
| Gem 1480 | patient |
| NCPT 66 | patient |
| NCPT 133 | compost |
| NCPT 145 | silage |
| NRRL 163 | lung of chicken |
| AF 293 (CBS101355) | patient |
